# Circulating tumour DNA is a promising biomarker for risk stratification of central chondrosarcoma with *IDH1/2* and *GNAS* mutations

**DOI:** 10.1002/1878-0261.13102

**Published:** 2021-09-30

**Authors:** Iben Lyskjær, Christopher Davies, Anna‐Christina Strobl, Joanna Hindley, Steven James, Radhesh K. Lalam, William Cross, Geoff Hide, Kenneth S. Rankin, Lee Jeys, Roberto Tirabosco, Jonathan Stevenson, Genomics England Research Consortium, Paul O’Donnell, Paul Cool, Adrienne M. Flanagan

**Affiliations:** ^1^ Research Department of Pathology University College London UCL Cancer Institute London UK; ^2^ Medical Genomics Research Group University College London UCL Cancer Institute London UK; ^3^ Department of Histopathology Royal National Orthopaedic Hospital Stanmore UK; ^4^ Department of Musculoskeletal Imaging Royal Orthopaedic Hospital Birmingham UK; ^5^ North of England Bone and Soft Tissue Tumour Service Freeman Hospital Newcastle UK; ^6^ Newcastle Centre for Cancer Newcastle University UK; ^7^ Orthopaedic Department Royal Orthopaedic Hospital NHS Foundation Trust Birmingham UK; ^8^ Department of Orthopaedic Oncology and Arthroplasty Royal Orthopaedic Hospital NHS Foundation Trust Birmingham UK; ^9^ Genomics England Research Consortium, William Harvey Research Institute Queen Mary University of London UK; ^10^ Department of Radiology Royal National Orthopaedic Hospital Stanmore UK; ^11^ Robert Jones & Agnes Hunt Orthopaedic Hospital NHS Foundation Trust Oswestry UK; ^12^ Keele University UK

**Keywords:** chondrosarcoma, circulating tumour DNA, GNAS, IDH1, IDH2, prognosis

## Abstract

Chondrosarcoma (CS) is a rare tumour type and the most common primary malignant bone cancer in adults. The prognosis, currently based on tumour grade, imaging and anatomical location, is not reliable, and more objective biomarkers are required. We aimed to determine whether the level of circulating tumour DNA (ctDNA) in the blood of CS patients could be used to predict outcome. In this multi‐institutional study, we recruited 145 patients with cartilaginous tumours, of which 41 were excluded. ctDNA levels were assessed in 83 of the remaining 104 patients, whose tumours harboured a hotspot mutation in *IDH1*/*2* or *GNAS*. ctDNA was detected pre‐operatively in 31/83 (37%) and in 12/31 (39%) patients postoperatively. We found that detection of ctDNA was more accurate than pathology for identification of high‐grade tumours and was associated with a poor prognosis; ctDNA was never associated with CS grade 1/atypical cartilaginous tumours (ACT) in the long bones, in neoplasms sited in the small bones of the hands and feet or in tumours measuring less than 80 mm. Although the results are promising, they are based on a small number of patients, and therefore, introduction of this blood test into clinical practice as a complementary assay to current standard‐of‐care protocols would allow the assay to be assessed more stringently and developed for a more personalised approach for the treatment of patients with CS.

## Graphical abstract

In this multi‐institutional study, we detected the amount of circulating tumour DNA (ctDNA) in the blood of patients with chondrosarcoma. We recruited 145 patients, and ctDNA levels were assessed in patients with tumours carrying *IDH1*/*2* or *GNAS* mutations. We found that ctDNA levels offered a more accurate identification of high‐grade tumours than pathology and were additionally associated with poor prognosis.

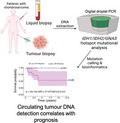

**Abbreviations**
ACT: atypical cartilaginous tumour
CS: central conventional chondrosarcoma
ctDNA: circulating tumour DNA
DDCS: dedifferentiated chondrosarcoma
ddPCR: digital droplet PCR
FFPE: formalin‐fixed paraffin‐embedded
G: grade
IDH: isocitrate dehydrogenase
RNOH: Royal National Orthopaedic Hospital
SNV: single nucleotide variant
WT: wild‐type

## 1 Introduction

Central conventional chondrosarcoma (CS) and dedifferentiated chondrosarcoma (DDCS) represent the most common primary bone tumours in adults. Clinical management and prognosis for this disease is largely determined by assessment of tumour grade and staging [1, 2] in the context of the anatomical site, and imaging of the primary tumour [2]. However, the interobserver variation in grading cartilaginous tumours by pathologists and radiologists demonstrates that these criteria are not reliable [3, 4]. Individual patient management would benefit from a more robust biomarker to predict relapse risk and survival, which could also be used for assessing response to treatment in clinical trials.

Well‐differentiated central cartilaginous tumours include enchondroma, atypical cartilaginous tumour (ACT), and CS grade (G) 1. ACT and CS G1 account for approximately 50% of central cartilaginous tumours (the incidence of enchondromas is unknown); they exhibit similar histological features and are distinguished on the basis of anatomical site, which determines clinical outcome and therefore management [2]. Well‐differentiated cartilaginous tumours occurring in the small tubular bones of the hands and feet and long bones have an excellent clinical outcome following curettage and are referred to as ACT [2]. Indeed, patients with CS G2 and G3 in the extremities rarely die of their disease because the risk of metastases is negligible [2, 5]. In contrast, well‐differentiated tumours presenting in the axial skeleton, pelvis, scapula, ribs and base of skull are referred to as CS G1 because of the high incidence of local recurrence at these sites even after attempted curative surgery [2]. A risk of transformation to a higher grade comes with incomplete excision [6].

CS G2 represents approximately 40% of central CS [7]. The majority of those affected will have major surgery involving *en bloc* excision, and when sited in the long bones, endoprosthetic reconstruction: the 5‐year survival ranges from approximately 70–99% [2, 7, 8, 9, 10]. By contrast, CS G3 – representing about 10% of all central CS – receive the same treatment as G2 disease but have a worse prognosis with a 5‐year survival ranging from 30% to 77% [7, 10]. Deaths continue to occur up to and beyond 10 years [7, 9]. Around 10% of all conventional chondrosarcoma become dedifferentiated, which is associated with a poor prognosis (7–24% 5‐year survival [11]). Variable clinical outcomes in tumours of the same grade are likely to be accounted for, at least in part, by the interobserver variability between cellular pathologists but also by differences in follow‐up, anatomical site and clinical management [7, 8].

In 2011, we reported that approximately 60% of central conventional and dedifferentiated CS harbour either an *IDH1* or an *IDH2* hotspot mutation [12, 13]. These mutations do not occur in peripheral chondrosarcomas, which are considerably less common than their central counterpart [2]. As *IDH1* and *IDH2* mutations occur early in the evolution of the disease [12, 14, 15, 16] and appear to be retained if the tumour transforms into higher grade disease [14], we considered that they could be exploited as biomarkers for clinical outcome. Other recurrent genetic events in CS, including *COL21A*, *CDKN2A* and *TP53*, do not occur as hotspot alterations and therefore represent less attractive biomarkers [13, 17]. Since the start of this project, *hTERT* canonical mutations have been identified as a marker of high‐grade disease [18, 19] and consequently was not a focus in this study.

Circulating tumour DNA (ctDNA) has shown promise as a minimally invasive tumour biomarker for many cancers in which it has predicted outcome and has been applied as a serial monitoring tool to detect early relapse [20, 21, 22]. Previously, we reported results from a small cohort of patients with central cartilaginous tumours (*n* = 29 CS) and showed that *IDH1‐* and *IDH2*‐mutant molecules (ctDNA) could be detected in plasma from all patients with CS G3 and dedifferentiated CS, in approximately 50% of those with G2 disease and never in patients diagnosed with well‐differentiated cartilaginous tumours [23]. Here, we report a multi‐institutional follow‐up study in which we employed a standardised protocol for screening *IDH1‐* and *IDH2*‐mutant molecules in circulating free DNA (cfDNA) from patients with CS. Furthermore, as central CS may occur in a small proportion of patients with fibrous dysplasia, a benign fibro‐osseous tumour caused by pathognomonic *GNAS* hotspot mutations [24], we assessed if detection of mutant *GNAS* molecules in plasma could also be employed in a similar manner to that of *IDH1/2‐*mutated ctDNA. We provide further evidence that screening for the presence of *IDH1‐* and *IDH2‐*mutant molecules in plasma from patients with central cartilaginous tumours leads to a more accurate grade and prognosis, when interpreted in the context of imaging and anatomical site, than currently provided.

## 2 Materials and methods

The project was registered as a NIHR portfolio study (CPMS ID – 35720 ‘Does circulating DNA predict the grade and disease burden of chondrosarcoma? A nationwide collaboration Study ctDNA and cartilaginous tumours’) approved by the Bloomsbury Research Ethics Committee: REC 17/LO/1423: IRAS project ID: 228173. Ethical approval was also obtained from the Research Tissue Bank UCL/UCLH Biobank ‘Studying Health and Disease from the Health Research’; National Research Ethics Committee reference 15/YH/0311. IRAS project ID: 183090. Written informed consents were obtained from the patients, and the study was conducted in accordance with the Declaration of Helsinki.

### 2.1 Study design

The aim was to enrol a minimum of 100 patients diagnosed with a central conventional or dedifferentiated cartilaginous tumours and 30 patients with enchondromas over 24 months from four bone tumour units in England and to follow all patients for a minimum of 12 months. The aims of the project were to determine whether (a) ctDNA in conjunction with imaging could diagnose chondrosarcoma without biopsy, (b) pre‐operative ctDNA levels indicated a poor‐prognosis, (c) whether ctDNA detection after surgery indicated residual disease and (d) whether serial monitoring of ctDNA detected disease relapse.

Children and patients with peripheral cartilaginous tumours were excluded. The age of the patient at the time of diagnosis of the cartilaginous tumour was defined as the date on which the tumour of interest was diagnosed, not the date on which the diagnosis of a syndrome, such as Ollier's disease or fibrous dysplasia, was made (Details of study protocol in Appendix S1).

The study was opened, and the first patient was enrolled in October 2017; the study was closed at the end of December 2020, which included a six‐month extension due to the COVID pandemic. However, 23 patients diagnosed prior to the start date for whom plasma samples had been biobanked were enrolled in the study (ethics ref: 15/YH/0311) (Table S1). Blood samples of 20 mL were to be taken prior to surgical treatment, at the first follow‐up appointment which occurred at approximately 6 weeks later, at regular intervals thereafter to coincide with outpatient appointments or if they were admitted to hospital between scheduled appointments (described in the protocol, see Appendix S1). Interim blood samples could also be taken by patients' General Practitioners; all samples were processed in the laboratory at the Royal National Orthopaedic Hospital (RNOH).

Tumour samples were processed and reported in the four diagnostic pathology laboratories according to their standard operating procedures. The formalin‐fixed paraffin‐embedded (FFPE) tumour biopsy and a representative block of the surgical specimen were sent to RNOH for review and DNA extraction. Grading was determined on the biopsy and reassessed on the surgical specimen.

DNA was extracted from 4 to 10 µm sections of FFPE tumour tissue (some were microdissected to avoid dilution with DNA from normal cells), using the QIAamp^®^ DNA FFPE Tissue kit (Qiagen, Hilden, Germany) as per the manufacturer’s instructions. 50 µL of elution buffer was passed through the column twice to increase the yield. For frozen sections, DNA was extracted from 20 to 60 µm sections using the QIAamp^®^ DNA Mini kit (Qiagen) as per the manufacturers’ instructions. 200 µL of elution buffer was passed through the column twice to increase the yield. DNA concentration was evaluated using a Qubit™ dsDNA HS Assay Kit (Thermo Fisher Scientific, Waltham, MA, USA) and a NanoDrop™ One Spectrophotometer (Thermo Fisher Scientific) and stored at −20 °C.

All samples were screened first for R132 *IDH1‐*mutations, and if not identified the samples were tested for R172 *IDH2*‐mutant molecules; R201C *GNAS*‐mutant molecules were sought in material from three patients whose CS arose on the background of fibrous dysplasia (Mazabraud syndrome) [25]. As the *IDH1* and *GNAS* droplet digital PCR (ddPCR) assays are susceptible to false positives from artefacts created by the deamination of cytosine in FFPE DNA [23], the DNA from FFPE samples was treated with uracil DNA glycosylase (Cat no. M0280, New England BioLabs, Ipswich, MA, USA) as per the manufacturer’s protocol prior to running their respective assays.

### 2.2 Processing of blood samples

Blood was received either in EDTA or PAX gene blood tubes (Qiagen). Samples in EDTA tubes were processed within 2 h of being drawn from the patient. Blood in PAX gene tubes was maintained at room temperature and processed within 10 days of being taken. The blood was centrifuged (VWR, Radnor, PA, USA) at 1600 ***g*** for 10 min at 4 °C, after which the plasma was decanted into DNA LoBind tubes (Eppendorf, Stevenage, UK). The tubes were then spun again (Thermo Fisher) at 1880 ***g*** for 10 min at 4 °C to remove remaining blood cells.

### 2.3 cfDNA extraction from plasma

Circulating free DNA (cfDNA) was extracted from 3 mL plasma per time point, using the QIAamp^®^ Circulating Nucleic Acid Kit (Qiagen) according to the manufacturer’s protocol. 55 µL of elution buffer was passed through the column twice to increase the yield. Once extracted, purified cfDNA was eluted and stored at −20 °C prior to use.

### 2.4 Droplet digital PCR (ddPCR) assays and analysis

cfDNA and tumour DNA were analysed on the QX200 Droplet Digital PCR System (Bio‐Rad, Hercules, CA, USA). 20 μL reactions consisted of up to 8.8 μL DNA, 10 μL 0.02x SuperMix for Probes (no dUTP; Cat. No 186‐3023, Bio‐Rad), 18 mm forward and reverse primers, 0.05 mm probe and nuclease‐free water. Droplets were generated on the QX100 Droplet Generator (Bio‐Rad), which then underwent 40 cycles of PCR (T100 Thermocycler, Bio‐Rad); 95 °C for 10 min, 44 cycles of 94 °C for 30 s, X °C for 1 min (X is assay specific; Table S2) prior to reading on a QX200 droplet reader (Bio‐Rad). Droplets were read with either the FAM/VIC or FAM/HEX channels setting provided by the quantasoft 1.7.4 software package (Bio‐Rad). Droplets were inspected visually and called as ‘mutant only’, ‘WT only’, ‘double‐positive’ or ‘template negative’. The presence of a detectable mutation in each tumour was verified in a multiplex experiment prior to the running of plasma samples, as previously reported [23]. A positive control, WT DNA and no‐template control were included in each run. The positive control was a patient’s sample with a mutation verified via whole genome analysis (100,000 Genomes Project [26, 27]), and the WT sample was a commercially available pooled sample of human placental DNA (BioLine, London, UK). The inclusion of the WT and no‐template control (H_2_O) in each assay run was used as a measure of background error rate. The plots were visualised in all cases. Each cfDNA sample was run in duplicate (8.8 µL DNA per run, 1 mL plasma analysed in total in two separate runs) on each assay run. Tumour and plasma samples were deemed to be mutant‐positive if they had a minimum of 2 ‘mutant‐only’ droplets with over 10 000 WT droplets also called. Samples with just a single mutant droplet were classified as equivocal and retested (2 mL plasma). For the plasma samples, mutant droplets were reported in terms of mutant copies per mL of blood plasma analysed. Blood samples were classified as containing circulating tumour DNA (ctDNA) if a minimum of two mutant‐only droplets were detected. Table S3 summarises all ddPCR results for the ctDNA‐positive cases. When available, postoperative plasma sample was tested for ctDNA from those patients that were pre‐operative negative for ctDNA, but who suffered a clinical relapse. In all instances, these plasma samples remained ctDNA negative. *IDH1* (*n* = 20) and *IDH2* mutations (*n* = 4) detected by ddPCR in this study were confirmed in the same tumours which had undergone whole genome sequencing as part of the 100,000 Genomes Project [27] (Table S1).

Specifically, both the mutant‐ and wild‐type probes, for the *IDH* and *GNAS* assays, were multiplexed in the same assay and this approach was used as a control to monitor the amount of DNA input into the assay. In the pilot study [23], we recognised that little DNA resulted in a weak or low level of our *IDH* wild‐type probe, whereas too much DNA changed the shape of the plot resulting in the assay being classified as ‘fail’. Therefore, the amount of plasma extracted (˜3 mL) was chosen so that > 95% of the samples would fall within the testable range. Any samples which failed were retested, however, only one case (ID99) required a lower DNA volume input in the pre‐op sample and notably this occurred in a patient who had suffered a pathological fracture which may have explained the finding.

Findings in our current study confirm our previous results in terms of interpreting ctDNA in patients with multiple enchondromas. This is illustrated by the following cases: ID140: a cartilage tumour in the radius from a patient with Ollier disease was diagnosed as an enchondroma following a needle biopsy and a decision was made not to treat. However, ctDNA was detected 60 days later just prior to curettage of an enchondroma in the phalanx. A postcurettage plasma was not available, but the disease in the affected arm and at other sites has not progressed (follow‐up > 4 years). ID126: ctDNA was not detected prior to the resection of the G1 chondrosarcoma of the scapula but postoperatively IDH1‐mutant molecules were detected. Neither this tumour nor other lesions have required treatment in the last 20 years since surgery.

Of the other 12 cases arising in Ollier disease or Maffucci syndrome, the tumours of interest were classified enchondromas (*n* = 2), G1 (*n* = 4), G2 (*n* = 5) and G3 (*n* = 1). ctDNA was detected pre‐operatively in three cases, all G2.

### 2.5 Radiology review

Imaging was reviewed by specialist musculoskeletal radiologists from the four units, each responsible for their own cases with no central or consensus review. Tumours were graded according to radiological features which covered pathology categories low grade/well‐differentiated (enchondroma/ACT/CS G1), and high grade disease was represented by CS G2/3 without distinction being made between these two grades; dedifferentiated (G4) CS, with distinction between low‐ and high‐grade appendicular lesions according to previously published criteria [28]. The tumours were measured in all three orthogonal planes using the standard PACS electronic callipers and the maximum dimension recorded. The radiologists were not aware of the histological grade. The date of imaging detection of recurrence, either local (based on MRI of the surgical site) or distant (based on plain film or CT imaging of the chest), was noted for each case.

### 2.6 Pathology review

The histology was classified using the WHO criteria [2] (2020) after which enchondromas, ACT and CS G1 were referred to as well‐differentiated cartilaginous tumours. All cases were reviewed independently by at least two pathologists (AMF and RT) blinded to the radiology and the original pathology reports. Where there was a disagreement, the cases were reviewed together and a consensus was reached (Table S1).

### 2.7 Statistical analysis

Statistical analysis was performed using the r statistical software[29]. Summary statistics were performed. Categorical variables were compared using Fisher’s exact test. A *P*‐value < 5% was deemed statistically significant. Survival analysis was performed using the Kaplan–Meier estimator with death as the end point. Overall survival was defined as time from diagnosis to disease‐related mortality or censured at the timepoint for the last follow‐up. Survival analysis utilised a standard Cox proportional hazard model, and multivariate testing was performed using ‘coxphf’ r package [30].

## 3 Results

### 3.1 Patient cohort and samples

**Fig. 1:**
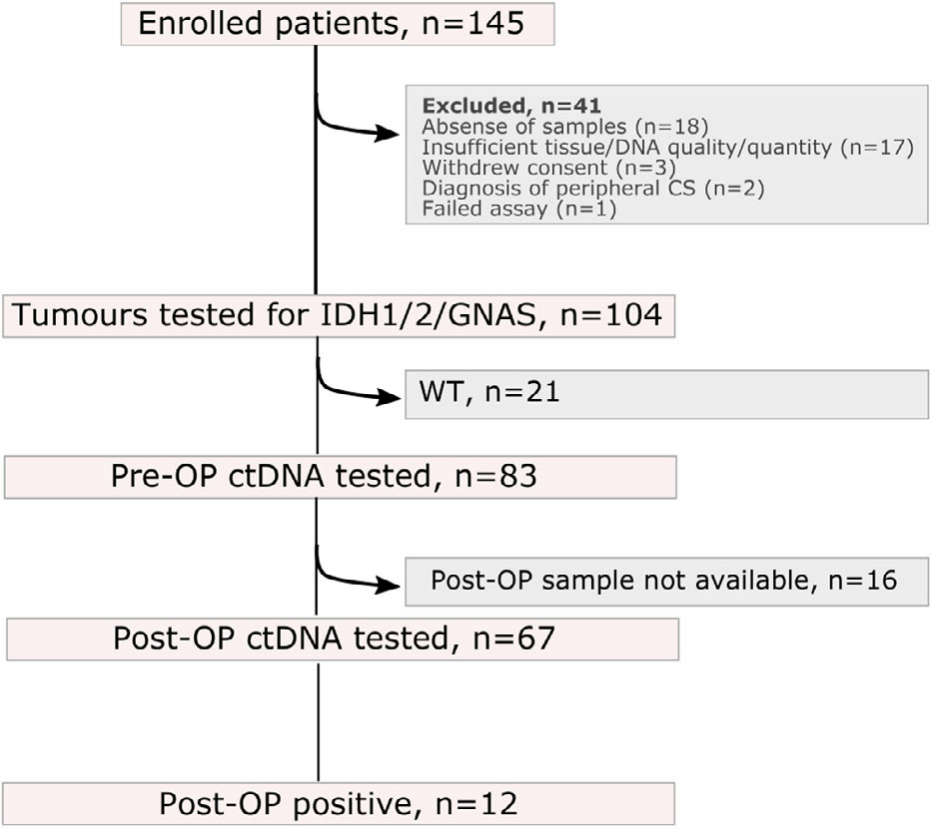
Study overview. The number of enrolled patients, and samples analysed. WT = wild‐type for *IDH1, IDH2, GNAS* hotspot mutations. Table S1 provides further details on each case.

145 patients were recruited to the study; 41 (28%) patients were excluded for a variety of reasons including three patients choosing to withdraw, diagnosis of a peripheral chondrosarcoma, absence of tissue submitted or inadequate DNA quality or quantity from plasma or histological material (Table S1). Mutational analysis was performed on the remaining samples from the 104 participants (72%) whose age at presentation ranged from 17 to 86 years (median age 53 years; 49 male, (47%) and 55 female (53%)). 21 patients (20%) were then excluded from further analysis as their tumours did not harbour a hotspot mutation of interest. The median follow‐up of the remaining 83 patients (80%), included in the ctDNA analysis, was 697 days (range 7–7558 days): they were aged between 17 and 86 years (median 55 years; 41 male (49%), 42 female (51%)). Figure 1 provides an overview of the study, with a breakdown of tumour grade in Table S1.

### 3.2 Radiology and pathology correlation

There was disagreement between the grading of pathology biopsies and radiology in 24 cases (17%, 121/145 recruited patients) (Tables S1 and S4). However, it is noteworthy that in 11 of these 24 cases the radiologists reported high‐grade disease (10 dedifferentiated CS and one grade 2 CS) which were reported by pathologists as well‐differentiated tumours. These discrepancies highlight the problem of tumour heterogeneity and nonrepresentative sampling on biopsy and underscore the need for multidisciplinary meetings.

Disagreement between pathologists was infrequent (5%, *n* = 7) and was mainly in the differentiation of enchondromas from ACTs, and in tumours sited in the bones of the extremities and therefore had little clinical impact (Table S1).

### 3.3 *IDH1* and *IDH2* hotspot mutations detected in 80% of tumours

Of the 104 tumours studied, 14 patients had multiple enchondromas (13 Ollier disease and one Maffucci syndrome), and three arose in patients with fibrous dysplasia (ID3, 24 and 38). A hot spot (recurrent) single nucleotide variant (SNV) of interest was detected in 83/104 (80%). 70 of the mutations were *IDH1* and 10 were *IDH2*, excluding one *IDH2* case with a pArg172lle mutation identified on whole genome sequencing; the *IDH1* and *IDH2* mutations were mutually exclusive. The remaining three patients harboured the R201 *GNAS* mutation characteristic of fibrous dysplasia. One of these tumours (ID3) also harboured the rare *IDH2* pArg172lle mutation which our ddPCR assay was not designed to detect: the other two tumours (ID24 and ID38) were WT for *IDH1* and *IDH2* mutations. 21/104 (20%) tumours were wild‐type (WT) for hot spot *IDH1/2* mutations. Tables 1 and 2 provide details of tumour grade and *IDH1/IDH2/GNAS* mutation status.

**Table 1:** Central chondrosarcoma histological grade on resection specimen, genotype of tumour and ctDNA in 104 patients.

	*IDH1* (*n* = 70) 23 positive for pre‐operatively ctDNA	*IDH2* (*n* = 10)^a^ 5 positive for ctDNA	WT (*n* = 21) *IDH1/2/GNAS*	*GNAS* (*n* = 3)^b^ 3 positive for ctDNA
Well differentiated (*n* = 41)	33	3	6	
Grade 2 (*n* = 42)	26	4	11	1
Grade 3 (*n* = 4)	2	1	1	
Dedifferentiated (*n* = 16)	9	2	3	2

^a^ A tumour harbouring an *IDH2* mutation (pArg172lle) not detectable by dPCR harboured a *GNAS* R201C mutation. This case is not included in the group.

^b^ Two tumours WT for *IDH1* and *IDH2* harboured a GNAS R201C mutation.

**Table 2:** Overview of correlation of tumour grade, *IDH1, IDH2* and *GNAS* mutant profile pre‐ and postoperatively.

	pre‐OP, *n* = 83		post‐OP, *n* = 69	
	ctDNA neg, *n* = 52	ctDNA pos, *n* = 31	ctDNA neg, *n* = 56	ctDNA pos, *n* = 13
Grade				
Well‐diff	35	2	27	0
High grade	17	21	29	8
Dediff	0	8	0	5
Genetic alteration				
*IDH1*	47	23	48	10
*IDH2*	5	5	7	1
*GNAS*	0	3	1	2
Max tumour size				
≤ 80 mm	41	5	37	2
> 80 mm	11	26	19	11

Max, maximum; Pre‐OP, pre‐operatively; Post‐OP, postoperatively.

### 3.4 Detection of pre‐operative ctDNA correlates with tumour grade

ctDNA was detected in pre‐operative plasma samples of 31/83 (37%) patients whose tumour harboured an *IDH1* or an *IDH2* mutation. The absence of ctDNA pre‐operatively correlated strongly with well‐differentiated tumours (*P* < 0.001, Fisher’s exact test), whereas its presence was strongly associated with both G3 and dedifferentiated CS (*P* < 0.001; Table 3). The association of G2 disease with detection of ctDNA was not statistically significant.

**Table 3:** Survival correlated with tumour grade, and detection of *IDH1, IDH2* and *GNAS* mutations in plasma pre‐ and postoperatively.

Survival (years)	Univariate analysis			Multivariate analysis^a^		
	HR	95% CI	*P*‐value	HR	95% CI	*P*‐value
Pre‐OP ctDNA (*n* = 83)						
Neg (*n* = 52)	Reference					
Pos (*n* = 31)	1.2*10^9^	0–Inf	**<0.0001**	60.2	2.4–10 373.7	**0.008**
Post‐OP ctDNA (69)						
Neg (*n* = 56)	Reference					
Pos (*n* = 13)	18.9	3.9–91.4	**<0.0001**	10.7	1.8–110.3	**0.007**
Grade (*n* = 83)						
Well‐diff (*n* = 36)	Reference					
High grade (*n* = 37)	5	0.6–42.9	0.14			
Dediff (*n* = 8)	41.5	4.9–348.5	**<0.001**	4.2	0.5–51.6	0.17
IDH status (*n* = 80)						
IDH1 (*n* = 70)	Reference					
IDH2 (*n* = 10)	1.5	0.1–5.0	0.7			
Gender (*n* = 83)						
Male (*n* = 41)	Reference					
Female (*n* = 42)	2.2	0.7–7.5	0.2			
Age (*n* = 83)						
< 55 years (*n* = 41)	Reference					
≥ 55 years (*n* = 42)	0.5	0.6–7.0	0.2			
Max tumour size						
≤ 80 mm	Reference					
> 80 mm	15.3	0.1–2.0	**0.01**	0.9	0.2–9.4	0.9

CI, confidence interval; HR, Hazard ratio; Post‐OP, postoperatively; Pre‐OP, pre‐operatively.

^a^ For the multivariate analysis, the variables used were those that were significant in the univariate analysis.

ctDNA was detected in only one of 37 subjects with a well‐differentiated tumour. This patient had Ollier disease (ID140) (described in Section 3.5). In contrast, ctDNA was detected pre‐operatively in 10/13 dedifferentiated CS, and 3 of 4 cases with G3 CS diagnosed on histology. The positive association of high‐grade CS with ctDNA is strengthened by the detection of ctDNA in 6/7 patients diagnosed with well‐differentiated disease on biopsy but where the diagnosis was amended to high‐grade disease in the resected specimen (ID10, 38, 40, 54, 58, 141) (Table S4). Notably, these tumours were recognised as high‐grade disease by the radiologists uninformed of the ctDNA results.

### 3.5 ctDNA is associated with tumour volume

ctDNA was not detected postoperatively in 2/15 patients (ID27, ID132) with metastases. However, in both cases lung metastases presented after wide excision of the primary tumour; they were single lesions and both measured < 40 mm.

ctDNA was detected pre‐operatively in 13/14 patients with dedifferentiated CS. The exception was patient ID99, who presented originally with a pathological fracture of the proximal femur at the site of a G2 tumour and was treated with resection and endoprosthetic reconstruction: ctDNA was not available before this first surgical procedure. ctDNA was also not detected in plasma taken at the time of the local recurrence 242 days following the first surgery. The excised recurrent tumour was 60 mm in maximum dimension and included a dedifferentiated component, measuring 30 mm.

**Fig. 2:**
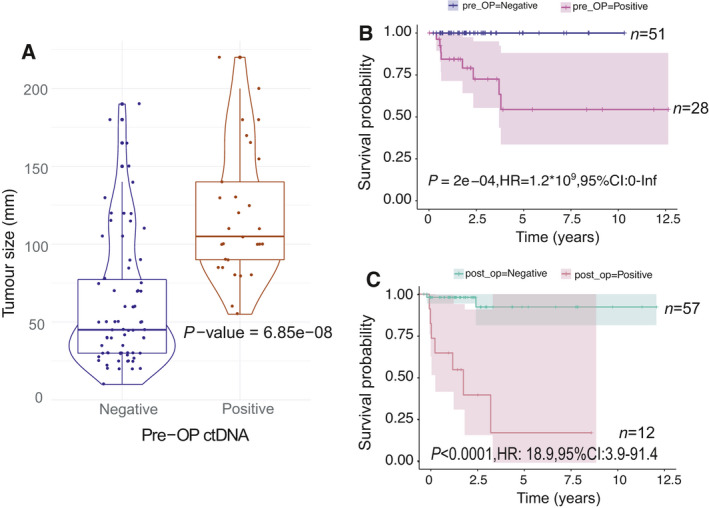
Detection of ctDNA pre‐ and postoperatively correlates with the risk of relapse. (A) ctDNA detection correlates with tumour size. (B) Detection of ctDNA pre‐operatively and (C) postoperatively correlates with overall survival (disease‐related mortality or censorship). Survival analysis utilised a standard Cox proportional hazard model.

Analysis of the minimum tumour size that was associated with metastasis in the 83 cases studied was 80 mm. Furthermore, we identified that detection of ctDNA pre‐operatively was related to larger tumour size (*P* < 0.001; Fig. 2A, Table 3).

### 3.6 ctDNA detection and multiple enchondromas: interpret with caution

We have previously reported that detection of ctDNA can occur post‐treatment of well‐differentiated tumours in the setting of multiple enchondromas, albeit in a small number of cases [23]. The three patients in question in the previous publication have not relapsed subsequently suggesting that detection of ctDNA should be interpreted with caution in this clinical setting. Findings in our current study confirm our previous results, as illustrated by the ID140 and ID126 (Appendix S1). Of the other 12 cases arising in Ollier disease or Maffucci syndrome, the tumours of interest were classified as two enchondroma (*n* = 2), four G1 (*n* = 4), five G2 (*n* = 5) and one G3 (*n* = 1). ctDNA was detected pre‐operatively in three cases, all G2.

### 3.7 Failure to detect ctDNA prior to surgery correlates with a good prognosis and anatomical site

Pre‐operative ctDNA was not detected in 51/81 (63%) patients, and notably ctDNA was never detected postoperatively if not detected pre‐operatively (plasma available in 41/51 of this group). None of these 51 patients developed systemic disease; tumours from 16 of these patients occurred in the bones of the hands and feet, with a maximum dimension of 60 mm (median = 30, range = 10–60 mm), and nine recurred locally. Three other tumours recurred including the following: ID81 – G2 femoral CS in the setting of Ollier disease in which *en bloc* excision with clear margins could not be achieved because of the extent of the lesion; ID70 – ACT of the distal femur which recurred locally twice following curettage, after which the tumour was resected *en blo*c: the patient has no evidence of relapse after 728 days. The final case (ID99) is described in Section 3.4.

Long‐term follow‐up is required to determine the predictive value of the absence of ctDNA detection pre‐operatively for tumours at anatomical sites other than the bones in the hands and feet as there is good evidence that there is negligible risk of metastatic disease when occurring at these sites [2, 15].

### 3.8 Detection of ctDNA correlates with the risk of relapse

ctDNA for *IDH1/2* and *GNAS* mutations was detected pre‐operatively in a total of 30/81 (37%) patients harbouring the relevant hot spot mutations in their tumours. The number of mutant molecules in these patients did not reflect tumour grade or tumour size. Of the postoperative plasma samples analysed from 69/81 patients (85%), mutant molecules were detected in 12 (15%) patients. The detection of ctDNA pre‐ and postoperatively correlates with survival (*P* < 0.001, Fig. 2B; *P* < 0.001, Fig. 2C, respectively, Table 3 **)**. Furthermore, the number of molecules were significantly reduced postoperatively compared with pre‐operative levels reflecting the tumour burden (*P* = 0.04).

ctDNA was detected pre‐operatively in all 11 patients in whom it was also detected postoperatively and all suffered a relapse; seven died of their disease, six with metastases (ID, 89, 54, 38, 17, 40, 28) and patient ID34 died of massive local disease involving the aorta. Two additional patients developed metastatic disease: ID103 whose lung was resected is alive without disease 9 years post‐thoracotomy. The second patient ID63 is alive with lung metastases. Two other patients (ID18 vertebra G2; ID112 pelvis G2) have persistent local disease following extensive surgery. The final patient (ID24 humerus, dedifferentiated) suffered a local recurrence (120 mm maximum dimension) which was treated with a wide local excision and has been event‐free for 514 days; no plasma has been obtained since the local recurrence.

Whereas ctDNA was detected postoperatively in all patients who suffered a relapse, of those in whom ctDNA was only detected pre‐operatively, 5/14 (36%) patients relapsed (ID3 sacrum, ID69 sacrum, ID27 femur, ID132 femur, ID91 femur). Of the remaining nine patients, five are at significant risk of relapse, notwithstanding the major surgery undertaken with intent to cure, because of the pelvic/sacral location. Another four patients (ID58, 78, 129, 42) with tumours in the long bones, two with dedifferentiated CS and two with G2 CS, remain disease‐free, but the follow‐up period is less than 36 months for these patients. Although relapse occurs in the vast majority of cases within 24 months of the primary surgery, relapse may occur up to a decade later as highlighted in patients ID3, ID27 and ID69 [7, 9].

### 3.9 ctDNA can detect relapse earlier than imaging

**Fig. 3:**
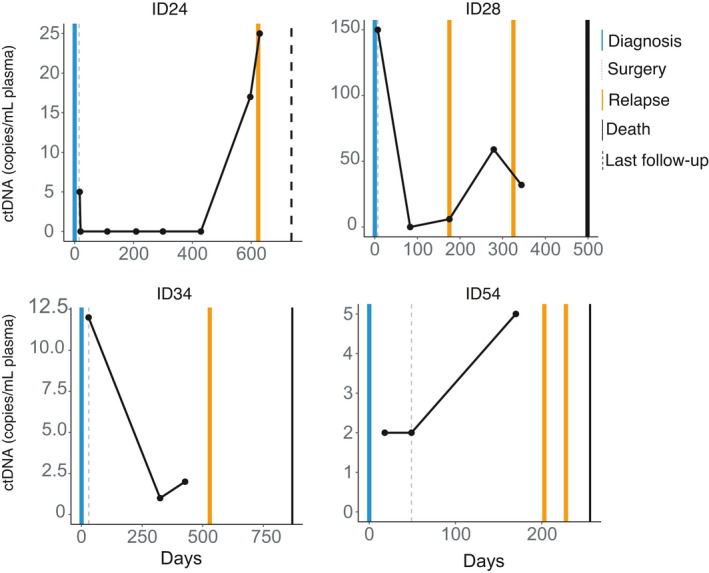
Schema of longitudinal ctDNA assessment showing detection prior to clinical relapse. Blue lines indicate time of diagnosis, grey dotted lines indicate time of surgery, orange lines depict clinical/radiological relapse, black filled lines indicate the time of death, and the dotted black lines represent the time of the last follow‐up. Two post‐OP ctDNA‐negative patients died (ID3 and 91); ID3 died of other causes, while ID91 died of the disease. Poor sampling in the follow‐up might explain the lack of detection of ctDNA in this patient postoperatively.

Of the 12 patients in whom ctDNA was detected both pre‐ and postoperatively, relapse was predicted by ctDNA prior to disease being detected clinically or on imaging in four cases (33%). ID34: 142 days ahead of imaging and 205 days ahead of the diagnosis being confirmed on histology. ID28: 150 days ahead of histology and imaging. ID54: 58 days ahead of imaging. ctDNA was detected in ID24 32 days prior to a local recurrence. In the remaining 8/12 patients, ctDNA was detected at the time of clinical detectable relapse (Fig. 3, Table S1). ctDNA was detected on the day on which clinical relapse was detected in another four patients (ID18, 40, 89, 103). In the last four patients, ctDNA was detected following relapse had been detected on radiology but prior to surgery for their metastasis/local recurrence (ID17, 38, 63, 112).

## 4 Discussion

Histological grading and imaging of cartilaginous tumours predict clinical behaviour unreliably; clinical and radiological surveillance are insensitive for detection of disease recurrence and are laborious and inefficient, particularly in patients with large metal implants. Therefore, the identification of more sensitive and accurate biomarkers would allow patients to receive a more personalised treatment plan. In this multicentre study, we have confirmed our previous findings [23] that digital droplet PCR mutation‐specific *IDH1*/*2* assays can be employed for the detection of ctDNA and we also report here for the first time that *GNAS* hot‐spot mutations, as with *IDH1/2* mutations, can be employed as a biomarker. Specifically, we have shown that (a) ctDNA in conjunction with imaging allows diagnosis of chondrosarcoma without biopsy, (b) that pre‐operative ctDNA levels indicate a significantly less favourable prognosis compared with those in which ctDNA was not detected, (c) that ctDNA detection after surgery indicates residual disease with the exception of patients with multiple enchondromas and (d) that serial monitoring of ctDNA detects disease relapse earlier than it is detected using current surveillance protocols. Although the results are promising and the specificity of the assay is underscored by the failure to detect mutant molecules of interest in the plasma of individuals with well‐differentiated tumours, and cartilaginous tumours of any grade in the extremities, with the exception, as previously reported [23], of patients with multiple enchondromas, the numbers of cases studied are small. Therefore, we would advocate for the assay to be introduced into clinical practice alongside existing standard‐of‐care protocols for providing diagnoses and prognoses. This approach would allow the test to be rigorously assessed and for confidence to be built in this blood‐based assay.

A considerable benefit of a blood sample, over current standard‐of‐care imaging and biopsy performed for the purpose of diagnosis and tumour grading, is that it overcomes the challenge of tumour heterogeneity and the potential for failing to sample the high‐grade component of the disease which has a significant impact on clinical management [6]. With our available data, we consider that this test would be a valuable adjunct to current standard‐of‐care testing but it would be ill‐advised at present to replace a diagnostic biopsy. It is also noteworthy that tumours other than cartilaginous neoplasms can harbour the same *IDH1/2* mutations assessed in our study, including carcinomas (predominantly cholangiocarcinoma), acute myeloid leukaemia and brain tumours although these mutations have never been identified in other primary bone tumours [12, 13, 31]. These findings highlight the importance of interpreting pathology in the context of the relevant imaging.

Albeit in only four patients, the ctDNA assay detected relapse earlier than currently used follow‐up methods: this small number is potentially related to the irregularity of the plasma sampling postoperatively and also the relatively short duration of this study. Although most relapses occur within 24 months of the diagnosis of chondrosarcoma, late relapses are well described [9]. However, if the assay was found to detect early relapse more commonly, it could potentially be useful for clinical surveillance in conjunction with MRI scans for chondrosarcoma, which are usually only performed when there is clinical concern of a local recurrence, by which time disease may be extensive and potentially unresectable. The absence of regular plasma samples may also have impacted on the ability to detect minimal residual disease. However, as the burden of disease appears to determine whether ctDNA is detected, the failure to detect ctDNA is best regarded as an ‘uninformative’ result whereas a positive result warrants further investigations and or close surveillance. Although compounded in this study by the COVID‐19 pandemic, we consider that taking blood samples for the measurement of ctDNA will remain a challenge until the practice of plasma collection is introduced as standard of care.

Major limitations of this study include the relatively small number of patients recruited, which is largely explained by the rarity of the disease. However, this number was compounded by the exclusion of 41 patients, 28% of those recruited because of poor quality or inadequate DNA, in addition to which 20–30% of chondrosarcomas which do not harbour an *IDH1/2* alteration [12]. The quality of DNA should not have such a negative impact on molecular testing today. The Royal College of Pathologists and WHO guidelines recommend decalcification protocols for bone samples; ideally biopsies should be decalcified in EDTA and some tissue from resection specimens should be set aside for decalcification in EDTA, and not in nitric or formic acid, as this allows DNA of sufficient quality to be extracted and used successfully for molecular tests, including droplet digital PCR [2, 32]. The benefit of optimising the decalcification proformas across all bone tumour sites would be substantial, as molecular assays are now commonly used for diagnosing not only primary bone tumours but also metastatic disease to bone. The 20–30% of central cartilaginous tumours without an *IDH1/2* mutation highlights the need for additional prognostic biomarkers for patients whose tumours are WT for *IDH1*, *IDH2* and *GNAS* mutations.

## 5 Conclusions

Implementation of this assay into clinical practice is the major challenge now faced. To provide robust evidence that this assay improves the health care of patients, ideally with cost–benefit, it will be necessary to build a large cohort of patients with CS and obtain plasma samples regularly with long‐term follow‐up. However, to ensure high level quality control of the testing with the relevant expertise, the assay would be best conducted in centralised laboratories.

The benefits of ‘liquid biopsies’ have been shown in a variety of the more common cancers such as breast [33], lung [21] and colorectal [20]. However, the benefits of such advances are likely to be delayed for patients with rare cancers because of the small numbers affected. Therefore, we argue that there is a case to be made to collect samples from patients with rare disease as part of routine care, so that they can be exploited in a timely manner for patient benefit.

## Conflict of interest

The authors declare no conflict of interest.

### Peer Review

The peer review history for this article is available at https://publons.com/publon/10.1002/1878‐0261.13102.

## Author contributions

AMF and POD designed the study; CD, ACS, POD, PC, JH, SJ, RKL, GH, KR, LJ, WC, RT, JS and Genomics England Consortium collected data; IL, PC, PO’D, CD and AMF analysed and interpreted the data. AMF and RT performed the pathology review; IL, AMF, PC and PO’D wrote the manuscript. All authors reviewed and edited the manuscript.

## Supporting information


**Table S1**. Clinical information.
**Table S2.** Digital droplet PCR assays.
**Table S3**. ctDNA results from the 31 patients with pre‐OP positive samples.
**Table S4**. Discrepancy between radiology assessment and pathology grading.

**Appendix S1**. Does circulating DNA predict the grade and disease burden of chondrosarcoma? A nationwide collaboration study.

## Data Availability

The data that support the findings of this study are available in the figures and in the supplementary material of this article.
