# Supplementary material for: Circulating tumour DNA is a promising biomarker for risk stratification of central chondrosarcoma with IDH1/2 and GNAS mutations
Source: Mol Oncol. 2021 Sep 30;15(12):3679–90. doi: 10.1002/1878-0261.13102 (PMC8637565; doi:10.1002/1878-0261.13102)
Supplement: Supplementary file 2 — Appendix S1. Does circulating DNA predict the grade and disease burden of chondrosarcoma? A nationwide collaboration study. [file MOL2-15-3679-s002.doc]

**Does circulating DNA predict the grade and disease burden of chondrosarcoma? A nationwide collaboration Study**

**Project Management Members**

1. **Chief Investigator:**

Professor Adrienne M Flanagan

Head of Department/Consultant

Histopathology, Institute of Orthopaedics, Royal National Orthopaedics Hospital

Brockley Hill, Stanmore

Middlesex

HA7 4LP

Tel. No: 020 8909 5354

Email: adrienne.flanagan@rnoh.nhs.uk

1. **Co-investigators:**

Mr Craig Gerrand

Consultant Orthopaedic Surgeon and Co-Clinical Director

Department of Orthopaedics
Freeman Hospital
Newcastle Upon Tyne
NE7 7DN

Tel. No: 01912 851651

Email: [Craig.Gerrand@nuth.nhs.uk](mailto:Craig.Gerrand@nuth.nhs.uk)

Professor Lee Jeys

Orthopaedic surgeon

Orthopaedic Department,

Royal Orthopaedic Hospital NHS Foundation Trust

Bristol Road South

Birmingham

B31 2AP

Tel. No: 0121 685 4000

Email: [lee.jeys@nhs.net](mailto:lee.jeys@nhs.net)

1. **Statistician:**

Mr Paul Cool

Orthopaedic Surgeon and Statistician

Robert Jones & Agnes Hunt Orthopaedic Hospital NHS Foundation Trust

Oswestry

SY10 7AG

Tel: 01691 404000

Email: [Paul.cool@nhs.net](mailto:Paul.cool@nhs.net)

1. **Project Manager**

Dr Anna Strobl PhD

Histopathology, Institute of Orthopaedics, Royal National Orthopaedics Hospital

Brockley Hill, Stanmore

Middlesex

HA7 4LP

Tel No: 02089095347

Email:

1. **Key Contact:**

Histopathology Research and Biobank Office,

Institute of Orthopaedics, Royal National Orthopaedics Hospital

Brockley Hill, Stanmore

Middlesex

HA7 4LP

Tel No: 02089095347

Email:

**Collaborators**

**Collaborator 1**

Name:    Kenneth Rankin

Department: Department of Orthopaedics

Institution: Freeman Hospital

Address: Newcastle Upon Tyne

Post Code: NE7 7DN

Tel. No: 01912 851651  Email: [Kenneth.Rankin@newcastle.ac.uk](mailto:Kenneth.Rankin@newcastle.ac.uk)

**Collaborator 2**

Name:    Dr Fernanda Amary

Department: Histopathology

Institution: Royal National Orthopaedic Hospital

Address: Brockley Hill, Stanmore, Middlesex

Post Code: HA9 4PL

Tel. No: 020 8909 5528  Email: [fernanda.amary@nhs.net](mailto:fernanda.amary@nhs.net)

**Collaborator 3**

Name:    Dr Paul O’Donnell

Department: Radiology

Institution: Royal National Orthopaedic Hospital

Address: Brockley Hill, Stanmore, Middlesex

Post Code: HA9 4PL

Tel. No: 07779 699051 Email: p.o'donnell@ucl.ac.uk

**Collaborator 4**

Name:   Dr Sumathi Vaiyapuri

Department: Musculoskeletal Pathology

Institution: Royal Orthopaedic Hospital NHS Trust

Address: Bristol Road South, Northfield, Birmingham

Post Code: B31 2AP

Tel. No: 0121 4147641 Email: [vaiyapuri.sumathi@nhs.net](mailto:vaiyapuri.sumathi@nhs.net)

**Collaborator 5**

Name:        Dr Prudencia Tyrell

Department: Radiology

Institution: Robert Jones & Agnes Hunt Orthopaedic Hospital NHS Foundation Trust

Address: Oswestry

Post Code: SY10 7AG

Tel. No: 01691 40400 Email: [Prudencia.Tyrrell@rjah.nhs.uk](mailto:Prudencia.Tyrrell@rjah.nhs.uk)

**Collaborator 6**

Name:      Dr Petra Dildey

Department: Histopathology

Institution: Newcastle Hospitals NHS Trust

Address: Freeman Hospital, Freeman Road, High Heaton

Newcastle upon Tyne

Post Code: NE7 7DN

Tel. No: 01912 829141 Email: [Petra.Dildey@nuth.nhs.uk](mailto:Petra.Dildey@nuth.nhs.uk)

**Collaborator 7**

Name:   Dr Roberto Tirabosco

Department: Histopathology

Institution: Royal National Orthopaedic Hospital

Address: Brockley Hill, Stanmore, Middlesex

Post Code: HA9 4LP

Tel. No: 0208 9095311 Email: [roberto.tirabosco@nhs.net](mailto:roberto.tirabosco@nhs.net)

**Collaborator 8**

Name:   Mr Paul Cool

Department: Histopathology

Institution: Robert Jones & Agnes Hunt Orthopaedic Hospital NHS Foundation Trust

Address: Oswestry

Post Code: SY10 7AG

Tel. No: 01691 404000 Email: [paul.cool@nhs.net](mailto:paul.cool@nhs.net)

**Collaborator 9**

Name:   Mr William Aston

Department: Bone Tumour Surgery

Institution: Royal National Orthopaedic Hospital

Address: Brockley Hill, Stanmore, Middlesex

Post Code: HA9 4LP

Tel. No: 020 8909 5619 Email: [jane.moody@rnoh.nhs.uk](mailto:jane.moody@rnoh.nhs.uk)

**Collaborator 10**

Name:   Mr Tom Cosker

Department: Orthopaedic Surgery

Institution: Oxford University Hospitals NHS Foundation Trust

Address: Nuffield Orthopaedic Centre, 7777 Old Road, Headington, Oxford, Oxfordshire

Post Code: OX3 7HE

Tel. No: 0300 304 7777 Email: tomcosker@hotmail.com

**Collaborator 11**

Name:   Dr Rajexh Botchu

Department: Department of Radiology

Institution: Royal Orthopaedic Hospital

Address: Birmingham

Post Code: B15 2QQ

Email: [rajesh.botchu@nhs.net](mailto:rajesh.botchu@nhs.net)

**Collaborator 12**

Name:   Professor Nicholas Athanasou

Department: Histopathology

Institution: Nuffield Orthopaedic Hospital

Address: Windmill Road, Oxford

Post Code: OX3 7LD

Email: [Nick.Athanasou@ouh.nhs.uk](mailto:Nick.Athanasou@ouh.nhs.uk)

**Collaborator 13**

Name:   Mr Rob Pollock

Department: Sarcoma Unit

Institution: Royal National Orthopaedic Hospital

Address: Brockley Hill, Stanmore, Middlesex

Post Code: HA9 4LP

Email: [Rob.Pollock@rnoh.nhs.uk](mailto:Rob.Pollock@rnoh.nhs.uk)

**Funder**

Bone Cancer Research Trust

10 Feast Field

Horsforth

Leeds

LS18 4TJ

**Sponsor**

Royal National Orthopaedic Hospital

Brockley Hill

Stanmore

Middlesex

HA7 4LP

Contents

[**1.** **INTRODUCTION 5**](#__RefHeading___Toc487470533)

[1.1 BACKGROUND 5](#__RefHeading___Toc487470534)

[1.2 RATIONALE FOR CURRENT STUDY 5](#__RefHeading___Toc487470535)

[2. STUDY OBJECTIVES 6](#__RefHeading___Toc487470536)

[2.1 PRIMARY AIM 6](#__RefHeading___Toc487470537)

[2.2 SECONDARY OBJECTIVE/S 6](#__RefHeading___Toc487470538)

[3. STUDY METHODOLOGY 6](#__RefHeading___Toc487470539)

[3.1 OVERALL DESIGN 6](#__RefHeading___Toc487470540)

[3.2 setting and timescale 7](#__RefHeading___Toc487470541)

[3.3 Study outcome measurements 8](#__RefHeading___Toc487470542)

[3.4 PARTICIPANTS 8](#__RefHeading___Toc487470543)

[3.5 RECRUITMENT AND METHODLOGICAL PROCESS 8](#__RefHeading___Toc487470544)

[4. ethical considerations 10](#__RefHeading___Toc487470545)

[5. ADVERSE EVENTS 11](#__RefHeading___Toc487470546)

[5.1 REPORTING PROCEDURES 11](#__RefHeading___Toc487470547)

[6. ASSESSMENT AND FOLLOW-UP 11](#__RefHeading___Toc487470548)

[7. STATISTICs and data analysis 11](#__RefHeading___Toc487470549)

[8. regulatory issues 12](#__RefHeading___Toc487470550)

[8.1 Ethics approval 12](#__RefHeading___Toc487470551)

[8.2 Consent 12](#__RefHeading___Toc487470552)

[8.3 Confidentiality 12](#__RefHeading___Toc487470553)

[8.4 Sponsor 12](#__RefHeading___Toc487470554)

[8.5 Funding & Costs 12](#__RefHeading___Toc487470555)

[8.6 Audits and Inspections 12](#__RefHeading___Toc487470556)

[9. Study Management 12](#__RefHeading___Toc487470557)

[10. Publication Policy 13](#__RefHeading___Toc487470558)

[11. References 13](#__RefHeading___Toc487470559)

# 1. INTRODUCTION

## 1.1 BACKGROUND

Conventional and dedifferentiated chondrosarcoma is the second most common primary bone tumour with an incidence of approximately 1/200,000 individuals/year. There are approximately 110 newly diagnosed chondrosarcomas annually in the UK (Whelan *et al*). There is evidence that the majority of these tumours arise from an enchondroma – a benign cartilaginous tumour. Chondrosarcoma are typically resistant to chemotherapy and radiotherapy and the overall survival rate has not changed in 30 years.

Clinical management decisions for patients with cartilage tumours are based on a combination of the tumour grade determined by a histopathologist based on its appearance down the microscope, and clinical evaluation – mainly imaging, and pain. Histological tumour grade (G) is the best prognosticator of metastatic disease and survival (apart from when the lesions occur in the phalanges as these have a negligible risk of metastasis). Broadly speaking, enchondroma and chondrosarcoma GI (low grade tumours) have an excellent prognosis and can be treated by curettage (scraping out the tumour from the bone). However approximately 15% of low grade cartilaginous tumour recur with some showing a high grade component at the time of recurrence (*Schawb et al; Ma et al*).

Chondrosarcoma GII and GIII (high grade disease) disease is generally treated with wide local excision and has a significantly higher risk of local recurrence and metastatic disease than enchondromas and GI tumours. However, the figures for survival vary between GII and GIII tumours: the 5 and 10 year survival rate of GII is 81% and 64%, and GIII is 43% and 29% respectively (*Fiorenza et al*). Patients who survive for 10 years are more likely to die from another disease.

Approximately 10% of conventional chondrosarcoma transform into a non-conventional variant known as dedifferentiated chondrosarcoma which are associated with a very poor 10% 5 year survival (*Bindiganavile et al*).

The variability in clinical outcome associated with grade is likely to be accounted for, at least in part, by the inter-observer variability between cellular pathologists in grading chondrosarcomas (*Eefting et al*). These challenges impact on clinical management of patients.

**Recent developments**

We were the first to show that *IDH1* mutations are detected in ~60% of conventional central chondrosarcomas and enchondromas (Amary *et al*). It is now known that the presence of *IDH1* mutations in the tumour neither affects the prognosis nor the clinical management of the patient.

We then investigated in a proof of concept study (in press Cancer Medicine) involving 30 patients if *IDH1* mutations can also be found in the blood , that is, as circulating tumour DNA (ctDNA). We found that 14 patients with GIII and dedifferentiated chondrosarcoma, and 50% of patients with GII chondrosarcoma (7 out of 14 patients) had measurable levels of mutant copies in their blood. Of note is that no circulating tumour DNA was identified in the blood of 8 patients with either an enchondroma or GI chondrosarcoma.

Based on our results **we hypothesise** that the detection of mutant *IDH1* in the blood is a more sensitive prognostic indicator for chondrosarcoma than histology.

## 1.2 RATIONALE FOR CURRENT STUDY

- Approximately 60% of central cartilaginous tumours have an *IDH1* mutation.
- Currently the most accurate prognostic indicator is the tumour grade (high and low grade), which is provided by the histopatholoists.
- Up to now treatment of cartilaginous tumours is largely based on histology: low and high grade tumours are treated differently with the latter being treated more radically and with greater morbidity.
- It is recognised that there is a lack of consistency and reproducibility between pathologists distinguishing between high and low grade cartilaginous tumours.
- We have preliminary evidence that we can detect ctDNA in the blood of patients with the high grade (GIII) cartilaginous, and in approximately 50% of patients with GII tumours but not in low grade cartilaginous tumours.
- Our preliminary data indicate that detection of pre-surgical ctDNA (mutant *IDH1*) is a more reproducible and accurate prognostic indicator of tumour behaviour than tumour grading.
- A blood test which could be taken at a GP surgery for monitoring patients for relapse rather than imaging at a hospital would be better for patients and it would be more cost-effective, efficient than what is currently offered.

# 2. STUDY OBJECTIVES

## 2.1 PRIMARY AIM

1. We aim to provide a more accurate and highly reproducible prognosis for patients with chondrosarcoma by evaluating a biomarker which is present in the majority of conventional central cartilaginous tumours (enchondroma and chondrosarcoma).

PRIMARY OBJECTIVES

1. Does detection of pre-operative ctDNA indicate a poorer prognosis than if not detected?
2. Does detection of post-surgery ctDNA predict tumour relapse?
3. Does ctDNA detection after surgery indicate residual and or metastatic disease?

iii) Can ctDNA in conjunction with imaging allow diagnosis of chondrosarcoma without a biopsy?

iv) Can correlation of ctDNA with histology reduce the variation in grading of cartilaginous tumours provided by histopathologists?

v) Can correlation of ctDNA with imaging reduce the variation in grading of cartilaginous tumours provided by radiologists?

### 2.2 SECONDARY OBJECTIVE/S

1. Establish a UK-wide collaborative clinical research network for chondrosarcoma, a disease in which neither clinical trials nor new treatments are available.
2. Samples will be stored for future investigation for other biomarkers while development of tests in other cancers is optimized.

# 3. STUDY METHODOLOGY

## 3.1 OVERALL DESIGN

We will recruit a minimum of 100 patients newly diagnosed with central chondrosarcoma, and 30 patients with benign cartilaginous tumours (enchondroma). Patients will be requested to give a small sample of blood before surgery and at their follow-up routine hospital appointments for up to a minimum of 12 months. In other respects patients will receive standard of care for their tumour.

- All equipment used in this study is serviced annually as required by UKAS standards
- All tests are performed according to the standard operating procedures in the clinical / diagnostic department which is a UKAS-accredited laboratory.
- All staff are trained in the process and signed as being capable of doing the work before being allowed to undertake this work.

**Blood samples**

20 ml of blood is taken by venepuncture prior to surgery and when the patient attends their routine follow up appointments and if they attend at other times.

- The blood sample is put into 2 DNA-stabilising tubes which will be supplied by the Sponsor site.
- The blood samples, which are stable for up to 1 week at room temperature, will be sent in stamped addressed packages employing the appropriate safety standards to the sponsor site for processing.
- The blood will be dispatched within 72 hours of being taken from the patient.
- The blood sample **must not be placed** in the fridge or freezer if in a DNA-stabilising tube.
- Receipt of the blood sample is logged by the research/study team and it is processed immediately according to standard operating procedures.
- The plasma and blood cells are frozen in a -80^0^C freezer. The temperature of the freezer is monitored and is alarmed is there is a drop in temperature.


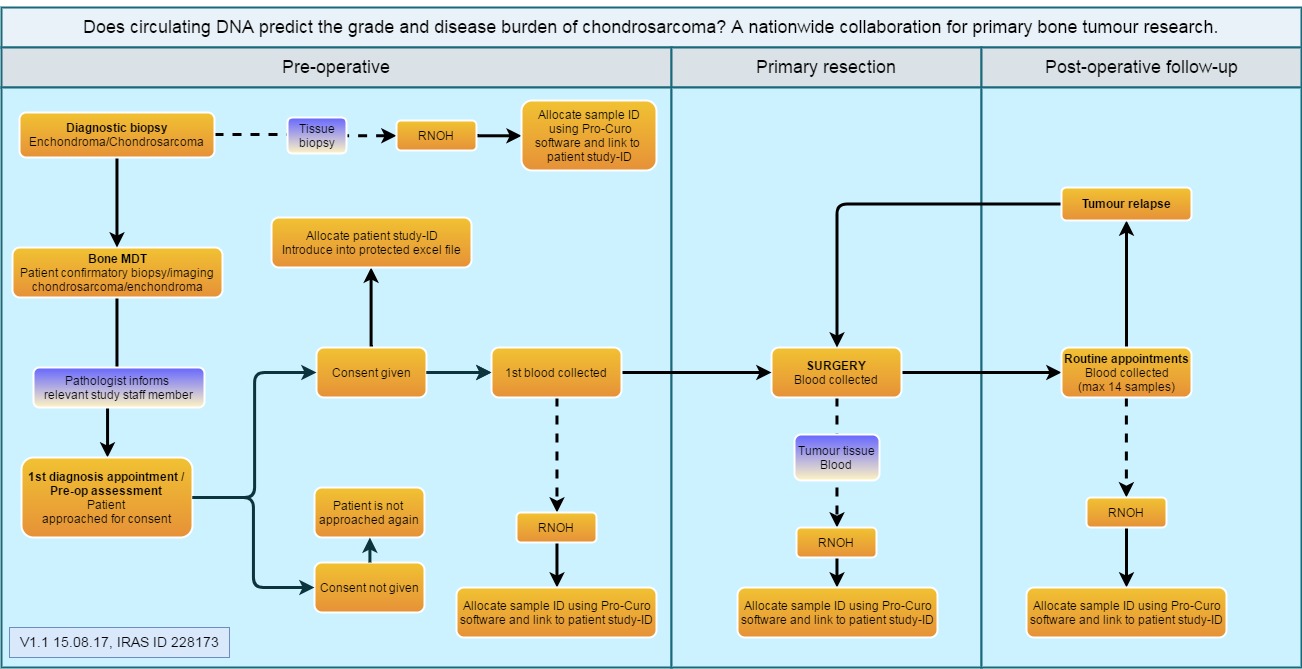


**Tumour samples**

The tumour biopsy and resection sample will be processed in the usual manner at each site and formal-fixed paraffin-embedded (FFPE) tissue will be produced. If there is tumour tissue which is surplus to diagnostic requirement it will be frozen and biobanked for future research projects at the hospital where the surgery takes place. The FFPE tissue treated in the normal manner and histology tissue sections are given to the histopathologist for a tissue diagnosis to be made. The result is discussed at the routine MDT meeting. Following the MDT meeting the tissue block is sent to the sponsor site (RNOH). The ‘sender’ site informs the sponsor site by secure email that the material has been despatched; on receipt of the material at the sponsor site, the research/study team informs the ‘sender’ of the sample arrival.

The sample with the required details is logged onto the study database (excel file) – see below.

The sponsor site cuts a tissue section, reviews the histopathology and then undertakes DNA extraction. The DNA will then be assessed for the presence of mutant *IDH1*. If an *IDH1* mutation is detected in the tumour, the plasma samples taken before and longitudinally after treatment will be tested for evidence of the mutant *IDH1*. In those patients whose tumours the *IDH1* mutation is not detected, ctDNA samples will be stored for future studies currently being developed.

**Measurement of IDH1 mutation**

Droplet digital polymerase chain reaction (ddPCR) is currently the most sensitive and accurate method for detecting and quantifying mutant DNA molecules. We will use the BioRad QX200 ddPCR platform for this study as using this machine we have demonstrated that we can detect the common *IDH1* mutations at below 1 mutant molecule in 10,000 wildtype molecules and quantify mutant molecules across over 4 orders of magnitude (Cancer Medicine in press). We have also optimized the ddPCR assays for the 7 common *IDH1* variants in both formalin-fixed paraffin-embedded tumour and blood samples.

Based on the background error rate of the assay (1 mutant only droplet per ~18,000 wild type droplets), and the average number of molecules we assessed per time point (n=1489), blood samples were deemed to be circulating tumour DNA (ctDNA)-positive if they had a minimum of 2 mutant only droplets.

This process is set up as a standard NHS clinical service with standard operating procedures in an UKAS-accredited histopathology department.

The equipment is serviced annually.

**Data Analysis**

The ctDNA level in the blood pre and post-surgery will be correlated with the tumour size, anatomical site, clinical outcome (symptoms, imaging for metastasis and local recurrence).

**Pathology and Radiology Review**

At least one representative pathologist and radiologist from each of the 5 different units will meet once every 9 months to review the pathology and radiology respectively from all patients. At least one representative surgeon from each of the 5 units will present their opinion as to the treatment that they would undertake. The opinions will be logged. At the end of the 36 months a meeting will take place to review all of the cases. This will improve the criteria employed for grading chondrosarcoma across the UK bone sarcoma centres.

## 3.2 setting and timescale

The patients will be recruited when they are being treated for a cartilaginous tumour in one of the 5 bone tumour units in England (Royal National Orthopaedic Hospital, Stanmore; Royal Orthopaedic Hospital Birmingham; Nuffield Orthopaedic Hospital, Oxford; Robert Jones and Agnes Hunt NHS Trust, Oswestry; and the Freeman Hospital Newcastle Upon Tyne.

Participants will be recruited from the start of the project for a 24-month period. All patients will be followed for a minimum period of 12 months. Hence the study will close at 36 months after its initiation.

| **Research Task** | **Time Points** | | | | |
| --- | --- | --- | --- | --- | --- |
|  | Appointment when patient given their diagnosis following biopsy and MDT meeting | Pre-op assessment | Surgery | 1^st^ post- op assessment (6 weeks after surgery) | Post–op assessments - national guidelines |
| Approach patients for participation (PIS given) |  |  |  |  |  |
| Consent obtained |  |  |  |  |  |
| Pre-op blood sample taken |  |  |  |  |  |
| Tissue obtained |  |  |  |  |  |
| Post-op blood sample obtained |  |  |  |  |  |
| Serial blood samples intervals at clinical appointments  or at GP surgery approximately every 3 months. Study ends at 36 months. |  |  |  |  |  |

Pre-op = before the operation/surgery

Post-OP = after the operation/surgery

BX = biopsy

## 3.3 Study outcome measurements

1. Detection of *IDH1* ctDNA pre-operatively
2. Levels of the *IDH1* ctDNA after surgery
3. Correlation of ctDNA levels with histology, radiology and clinical behaviour of tumour

## 3.4 PARTICIPANTS

**Groups**

In this study we will prospectively recruit a minimum of 100 patients newly diagnosed with chondrosarcoma and 30 patients with cartilaginous tumours (enchondroma – benign cartilage tumours).

**Inclusion criteria**

All patients 16 and above with a tissue and/or an imaging diagnosis of conventional and dedifferentiated chondrosarcoma, enchondroma, and atypical cartilaginous tumours (WHO criteria) identified at the bone tumour MDT meetings will be invited to participate in the study.

**Exclusion criteria**

Participants may not enter the study if any of the following apply;

- Prisoners
- Non-NHS patients
- Patients who are not capable of understanding the study
- Patients who are needle-phobic
- Patients who decline
- Patients know to hepatitis B or C positive; patients with HIV infection.

## 3.5 RECRUITMENT AND METHODLOGICAL PROCESS

**Recruitment**

- A tissue diagnosis of enchondroma/chondrosarcoma will be made by a consultant pathologist.
- Cases are discussed at the MDT meeting where the diagnosis is confirmed and the patient is identified as a possible candidates for the study. The MDT co-ordinator will then inform the research /study team (eg. Biobank staff or research nurses) about the eligible patients via secured email (nhs.net or trust intranet).
- The research/study team will make arrangements to approach the patient at the next routine hospital appointment (diagnosis appointment) to introduce him/her to the study and give the PIS.
- Consent to enter the study will be sought from each participant only after a full explanation about the study has been given, an information leaflet offered and time allowed for consideration.
- Consent is sought at a pre-op assessment appointment or prior to surgery at which time the first blood sample could be taken; alternatively the blood sample could be taken when the patient is under anaesthetic prior to the start of the operation.
  - - The Patient has the opportunity to decline consent. The consent form will be countersigned by the study/research team member; a copy of the signed form is given to the patient for their records.
- The research/study team will allocate the patients with a unique patient-ID (see below) on a password protected excel file (each participant site will have their own specific file).
- A blood sample will be obtained at each patient’s routine follow up hospital appointments for up to a minimum of 12 months or for the duration of the study (range 12 - 36 months).
- If routine appointments are not within 3 months, a letter will be sent to the patient for the attention of their GP surgery where patients may have their blood sample taken and sent to the sponsor site.

All members of the research/study teams will be trained in obtaining informed consent, safeguarding adults and research and information governance. The recruitment process will be the same across

all 5 sites.

**Tracking Patients and Anonymization**

1. **Creating a unique anonymised study ID for each patient:**
   After a patient has given written consent and agreed to take part in this study, the local at the recruitment site will inform the project co-ordinator at the Sponsor site. The project co-ordinator will allocate each patient with a unique study ID. Both individuals (the project co-ordinator at the Sponsor site and the administrator at the local sites) will record the unique study ID against the patient’s clinical details (name, DOB, NHS number, hospital site from which they were recruited, local hospital number) on their respective study logs. This unique study ID is a 4 digit number pre-fixed by the Hospital ID and the patient’s initials e.g. NOH_JS_0001**.** See Table below for hospital site ID.
2. The study log will be held on a NHS computer at all participating sites. The office in which the computer holding the log is held is secure: the computer and the study folder file will be password-protected. Apart from the project co-ordinator and the local administrators, the only other persons who will hold these passwords will be the R&D managers on the various NHS Trust sites: this is in case anything were to happen to the project co-ordinator. This ensures that researchers and individuals how take blood including the clinical team are not able to link the Unique study ID with data and samples.

All paper documents containing patient identifiable data will be kept in lockable storage investigator sites.

1. **Sample ID, linking to unique patient ID and sample tracking**All samples will be labelled at the study sites with the patient’s unique study ID (for example NOH_JS_0001) in addition to the date the sample was taken. The local investigators will document on the study log that a sample has been taken and the date on which it was taken and the date it was posted to the Sponsor site. When the sample is put in the post the local administrator will send an email stating that a sample is to be expected.

On receipt of the sample at the Sponsor site, the researchers will log it onto Pro-Curo, an established commercially available auditable software programme, used for tracking and barcoding samples. Each sample ID will be allocated an ID by Pro-curo in an automated fashion and this sample ID will be linked to the unique study ID of the relevant patient. All samples will then be labelled with the unique study ID (for example RNOH_JS_0001) plus the Pro-curo sample ID, thereby ensuring that the samples are completely anonymised to the research and clinical staff. This is because the researchers will not be able to link the patient unique ID or the sample IDs to the patient’s name, date of birth, hospital number and NHS number.

**Site Codes for Generation of Unique Patients’ IDs**

| **NHS Trusts - Sites** | **Site Code** |
| --- | --- |
| Royal National Orthopaedic Hospital | RNOH |
| Nuffield Orthopaedic Hospital | NOH |
| Newcastle Upon Tyne NHS Trust | NUT |
| Robert Jones and Agnes Hunt Orthopaedic Hospital NHS Trust | OSW |
| Royal Orthopaedic Hospital NHS Foundation Trust | ROH |

# 4. ethical considerations

1. All information about participants and samples will be treated in the strictest confidence.
2. The team involved in the study is trained in information governance.
3. Patient samples will be pseudo-anonymised so that researchers will not have direct access to patients’ identifiable data (see example of Patient ID below)
4. Patient Data will be tracked on a secure NHS web-based system accessible by password and access granted by sponsor.
5. The data will be backed-up using the standard NHS system in our UKAS-accredited laboratory in case there is an IT failure. This back up system will be checked for robustness on a regular basis.

# 5. ADVERSE EVENTS

The collection of research blood samples does not constitute an intervention that involves the administration of any medication to the participant. Therefore the risk of a serious adverse event is small. However unexpected event such as breakage of a blood tube or a reaction to a skin dressing may occur. Specific risks are listed below.

1. **Complications as a result of Venepuncture**

It is possible a patient may develop a haematoma as a part of normal venepuncture procedure. The member of staff taking the blood sample will be trained and signed off in venepuncture, infection control and health and safety.

1. **Loss of sensitive patient data**

All members of the study will have undertaken training in information governance, good clinical practice and have knowledge of the human tissue act thereby reducing the risk of data loss.

## 5.1 REPORTING PROCEDURES

In the event of adverse events, investigations will be held by the principal investigator at the site where the incident took place via normal Trust adverse event reporting protocols.

All incidences will be documented in the site file and reported to the study sponsor and the Chief Investigator.

# 6. ASSESSMENT AND FOLLOW-UP

Patients will be followed up according to national and local protocols, looking for local and systemic relapse within the requested funding envelope. We aim for a minimum of 12 months and a maximum of 36 months follow up. We anticipate that follow-up beyond this will become part of routine clinical practice.

# 7. STATISTICs and data analysis

Data and all appropriate documentation will be stored for a minimum of 5 years after the completion of the 36 month study.

The statistical analysis will be undertaken by an experienced statistician (Mr Paul Cool).

Kaplan-Meier survival analysis.

Cox proportional hazard analysis for factors associated with survival.

Genomic quantitative and qualitative data analysis to be undertaken by bioinformaticians and statisticians.

Apart from basic data visualization the data will be subjected to logistic regression analysis with the diagnosis as a categorical outcome variable and the circulating DNA levels (as well as other) as an independent continuous numerical variable. Depending on the result of the initial analysis appropriate threshold levels will be identified and the data will be subjected to Kaplan-Meier Aalen-Johansen survival analysis. The Cox proportional hazards model will be used to examine subsequently if circulating DNA (ctDNA) levels are associated with prognosis. Using sequential monitoring of ctDNA levels, time series will be visualised and analysed using standard prediction techniques and Auto-Regressive Integrated Moving Average (ARIMA) modelling.

# 8. regulatory issues

## 8.1 Ethics approval

The Chief Investigator has obtained approval from the xxx Research Ethics Committee. The study will be conducted in accordance with the recommendations for physicians involved in research on human subjects adopted by the 18th World Medical Assembly, Helsinki 1964 and later revisions.

## 8.2 Consent

Consent to enter the study must be sought from each participant only after a full explanation has been given, an information leaflet offered and time allowed for consideration. Signed participant consent should be obtained. The right of the participant to refuse to participate without giving reasons must be respected. A copy of the signed Informed Consent will be given to the participant. The original signed form will be retained at the study site. The person who obtained the consent must be suitably qualified and experienced, and have been authorised to do so by the Chief/Principal Investigator.

## 8.3 Confidentiality

The Chief Investigator will preserve the confidentiality of participants taking part in the study in line with the Data Protection Act 1998.

## 8.4 Sponsor

Royal National Orthopaedic Hospital

Brockley Hill

Stanmore

Middlesex

HA7 4LP

## 8.5 Funding & Costs

Bone Cancer Research Trust is funding this study.

## 8.6 Audits and Inspections

Regular audits and monitoring of the project will be undertaken by the sponsor and other regulatory bodies to ensure adherence to Good Clinical Practice and the NHS Research Governance Framework for Health and Social Care (2^nd^ edition).

# 9. Study Management

The day-to-day management of the study will be co-ordinated through a steering committee which will meet on a quarterly basis.

# 10. Publication Policy

The results of this study will be published in international peer reviewed journals. No patient identifiable data will be used in the published work resulting from this research.

# 11. References

Amary MF, Bacsi K, Maggiani F, Damato S, Halai D, Berisha F, Pollock R, O'Donnell P, Grigoriadis A, Diss T, Eskandarpour M, Presneau N, Hogendoorn PC, Futreal A, Tirabosco R, Flanagan AM. [IDH1 and IDH2 mutations are frequent events in central chondrosarcoma and central and periosteal chondromas but not in other mesenchymal tumours.](http://www.ncbi.nlm.nih.gov/pubmed/21598255) J Pathol. 2011 Jul;224(3):334-43.

Amary MF, Damato S, Halai D, Eskandarpour M, Berisha F, Bonar F, McCarthy S, Fantin VR, Straley KS, Lobo S, Aston W, Green CL, Gale RE, Tirabosco R, Futreal A, Campbell P, Presneau N, Flanagan AM. [Ollier disease and Maffucci syndrome are caused by somatic mosaic mutations of IDH1 and IDH2.](http://www.ncbi.nlm.nih.gov/pubmed/22057236) Nat Genet. 2011 Nov 6;43(12):1262-5.

Amary MF, Ye H, Forbes G, Damato S, Maggiani F, Pollock R, Tirabosco R, Flanagan AM. [Isocitrate dehydrogenase 1 mutations (IDH1) and p16/CDKN2A copy number change in conventional chondrosarcomas.](http://www.ncbi.nlm.nih.gov/pubmed/25432631) Virchows Arch. 2015 Feb;466(2):217-22. doi: 10.1007/s00428-014-1685-4.

Bettegowda C, Sausen M, Leary RJ, Kinde I, Wang Y, Agrawal N, Bartlett BR, Wang H, Luber B, Alani RM, Antonarakis ES, Azad NS, Bardelli A, Brem H, Cameron JL, Lee CC, Fecher LA, Gallia GL, Gibbs P, Le D, Giuntoli RL, Goggins M, Hogarty MD, Holdhoff M, Hong SM, Jiao Y, Juhl HH, Kim JJ, Siravegna G, Laheru DA, Lauricella C, Lim M, Lipson EJ, Marie SK, Netto GJ, Oliner KS, Olivi A, Olsson L, Riggins GJ, Sartore-Bianchi A, Schmidt K, Shih lM, Oba-Shinjo SM, Siena S, Theodorescu D, Tie J, Harkins TT, Veronese S, Wang TL, Weingart JD, Wolfgang CL, Wood LD, Xing D, Hruban RH, Wu J, Allen PJ, Schmidt CM, Choti MA, Velculescu VE, Kinzler KW, Vogelstein B, Papadopoulos N, Diaz LA Jr. [Detection of circulating tumor DNA in early- and late-stage human malignancies.](http://www.ncbi.nlm.nih.gov/pubmed/24553385) Sci Transl Med. 2014 Feb 19;6(224):224ra24.

Bindiganavile S, Han I, Yun JY, Kim HS. [Long-term Outcome of Chondrosarcoma: A Single Institutional Experience.](http://www.ncbi.nlm.nih.gov/pubmed/25687868) Cancer Res Treat. 2015 Oct;47(4):897-903.

Dawson SJ, Tsui DW, Murtaza M, Biggs H, Rueda OM, Chin SF, Dunning MJ, Gale D, Forshew T, Mahler-Araujo B, Rajan S, Humphray S, Becq J, Halsall D, Wallis M, Bentley D, Caldas C, Rosenfeld N. [Analysis of circulating tumor DNA to monitor metastatic breast cancer.](http://www.ncbi.nlm.nih.gov/pubmed/23484797) N Engl J Med. 2013 Mar 28;368(13):1199-209.

Diehl F, Schmidt K, Choti MA, Romans K, Goodman S, Li M, Thornton K, Agrawal N, Sokoll L, Szabo SA, Kinzler KW, Vogelstein B, Diaz LA Jr. [Circulating mutant DNA to assess tumor dynamics.](http://www.ncbi.nlm.nih.gov/pubmed/18670422) Nat Med. 2008 Sep;14(9):985-90.

Eefting D, Schrage YM, Geirnaerdt MJ, Le Cessie S, Taminiau AH, Bovée JV, Hogendoorn PC; EuroBoNeT consortium. [Assessment of interobserver variability and histologic parameters to improve reliability in classification and grading of central cartilaginous tumors.](http://www.ncbi.nlm.nih.gov/pubmed/18852676) Am J Surg Pathol. 2009 Jan;33(1):50-7.

Evans HL, Ayala AG, Romsdahl MM. [Prognostic factors in chondrosarcoma of bone: a clinicopathologic analysis with emphasis on histologic grading.](http://www.ncbi.nlm.nih.gov/pubmed/890662) Cancer. 1977 Aug;40(2):818-31.

Fiorenza F, Abudu A, Grimer RJ, Carter SR, Tillman RM, Ayoub K, Mangham DC, Davies [Risk factors for survival and local control in chondrosarcoma of bone.](http://www.ncbi.nlm.nih.gov/pubmed/11837841) AM. J Bone Joint Surg Br. 2002 Jan;84(1):93-9.

Forshew T, Murtaza M, Parkinson C, Gale D, Tsui DW, Kaper F, Dawson SJ, Piskorz AM, Jimenez-Linan M, Bentley D, Hadfield J, May AP, Caldas C, Brenton JD, Rosenfeld N. [Noninvasive identification and monitoring of cancer mutations by targeted deep sequencing of plasma DNA.](http://www.ncbi.nlm.nih.gov/pubmed/22649089) Sci Transl Med. 2012 May 30;4(136):136ra68.

Krumbholz M, Hellberg J, Steif B, Bäuerle T, Gillmann C, Fritscher T, Agaimy A, Frey B, Juengert J, Wardelmann E, Hartmann W, Juergens H, Dirksen U, Metzler M. [Genomic EWSR1 Fusion Sequence as Highly Sensitive and Dynamic Plasma Tumor Marker in Ewing Sarcoma.](http://www.ncbi.nlm.nih.gov/pubmed/27283964) Clin Cancer Res. 2016 Sep 1;22(17):4356-65.

[Ma XJ](http://www.ncbi.nlm.nih.gov/pubmed/?term=Ma XJ%5BAuthor%5D&cauthor=true&cauthor_uid=22009830), [Dong Y](http://www.ncbi.nlm.nih.gov/pubmed/?term=Dong Y%5BAuthor%5D&cauthor=true&cauthor_uid=22009830), [Zhang CL](http://www.ncbi.nlm.nih.gov/pubmed/?term=Zhang CL%5BAuthor%5D&cauthor=true&cauthor_uid=22009830), [Zeng BF](http://www.ncbi.nlm.nih.gov/pubmed/?term=Zeng BF%5BAuthor%5D&cauthor=true&cauthor_uid=22009830). Recurrence analysis in 66 cases with grade I and grade II chondrosarcomas in the extremities. [Orthop Surg.](http://www.ncbi.nlm.nih.gov/pubmed/22009830) 2009 May;1(2):132-6.

McBride DJ, Orpana AK, Sotiriou C, Joensuu H, Stephens PJ, Mudie LJ, Hämäläinen E, Stebbings LA, Andersson LC, Flanagan AM, Durbecq V, Ignatiadis M, Kallioniemi O, Heckman CA, Alitalo K, Edgren H, Futreal PA, Stratton MR, Campbell PJ. [Use of cancer-specific genomic rearrangements to quantify disease burden in plasma from patients with solid tumors.](http://www.ncbi.nlm.nih.gov/pubmed/20725990) Genes Chromosomes Cancer. 2010 Nov;49(11):1062-9.

[Schwab JH](http://www.ncbi.nlm.nih.gov/pubmed/?term=Schwab JH%5BAuthor%5D&cauthor=true&cauthor_uid=17514007)1, [Wenger D](http://www.ncbi.nlm.nih.gov/pubmed/?term=Wenger D%5BAuthor%5D&cauthor=true&cauthor_uid=17514007), [Unni K](http://www.ncbi.nlm.nih.gov/pubmed/?term=Unni K%5BAuthor%5D&cauthor=true&cauthor_uid=17514007), [Sim FH](http://www.ncbi.nlm.nih.gov/pubmed/?term=Sim FH%5BAuthor%5D&cauthor=true&cauthor_uid=17514007). Does local recurrence impact survival in low-grade chondrosarcoma of the long bones? Clin Orthop Relat Res. 2007 Sep;462:175-80.

Tarpey PS, Behjati S, Cooke SL, Van Loo P, Wedge DC, Pillay N, Marshall J, O'Meara S, Davies H, Nik-Zainal S, Beare D, Butler A, Gamble J, Hardy C, Hinton J, Jia MM, Jayakumar A, Jones D, Latimer C, Maddison M, Martin S, McLaren S, Menzies A, Mudie L, Raine K, Teague JW, Tubio JM, Halai D, Tirabosco R, Amary F, Campbell PJ, Stratton MR, Flanagan AM, Futreal PA. [Frequent mutation of the major cartilage collagen gene COL2A1 in chondrosarcoma.](http://www.ncbi.nlm.nih.gov/pubmed/23770606) Nat Genet. 2013 Aug;45(8):923-6

Whelan J, McTiernan A, Cooper N, Wong YK, Francis M, Vernon S, Strauss SJ. [Incidence and survival of malignant bone sarcomas in England 1979-2007.](http://www.ncbi.nlm.nih.gov/pubmed/21913189) Int J Cancer. 2012 Aug 15;131(4):E508-17.

*WHO Classification of Tumours of Soft Tissue and Bone, 4th edition, Chapter 15.* 2013.

**Appendices**

None
